# A stochastic model of size control in the budding yeast cell cycle

**DOI:** 10.1186/s12859-019-2839-9

**Published:** 2019-06-20

**Authors:** Mansooreh Ahmadian, John J. Tyson, Yang Cao

**Affiliations:** 1 Department of Computer Science, Virginia Tech, Blacksburg, VA USA; 2 Department of Biological Sciences, Virginia Tech, Blacksburg, VA USA

**Keywords:** Cell cycle, Size control mechanism, Budding yeast, Hybrid model, Stochastic model, Deterministic model, Stochastic size control, Cell cycle variability, G1 phase variability

## Abstract

**Background:**

Cell size is a key characteristic that significantly affects many aspects of cellular physiology. There are specific control mechanisms during cell cycle that maintain the cell size within a range from generation to generation. Such control mechanisms introduce substantial variabilities to important properties of the cell cycle such as growth and division. To quantitatively study the effect of such variability in progression through cell cycle, detailed stochastic models are required.

**Results:**

In this paper, a new hybrid stochastic model is proposed to study the effect of molecular noise and size control mechanism on the variabilities in cell cycle of the budding yeast *Saccharomyces cerevisiae*. The proposed model provides an accurate, yet computationally efficient approach for simulation of an intricate system by integrating the deterministic and stochastic simulation schemes. The developed hybrid stochastic model can successfully capture several key features of the cell cycle observed in experimental data. In particular, the proposed model: 1) confirms that the majority of noise in size control stems from low copy numbers of transcripts in the G1 phase, 2) identifies the size and time regulation modules in the size control mechanism, and 3) conforms with phenotypes of early G1 mutants in exquisite detail.

**Conclusions:**

Hybrid stochastic modeling approach can be used to provide quantitative descriptions for stochastic properties of the cell cycle within a computationally efficient framework.

## Background

Progression through eukaryotic cell cycle is governed by various control mechanisms such that new born progenies are able to repeat the cycle, while maintaining cell size in certain range generation after generation. Such complex control mechanisms are regulated by positive and negative feedbacks. The feedbacks create bistable switches to make the progression through different phases (G1 → S → G2 → M) irreversible. The specification of these biochemical feedbacks varies between different organisms. Extensive experimental studies have been carried out to identify the underlying molecular mechanisms that regulate the cell cycle [1–16]. Many signaling pathways in regulatory networks in addition to myriad of mutant phenotypes have been explored. Along with experimental studies, various mathematical models, including deterministic models, boolean networks, stochastic models, and hybrid approaches have been developed to quantitatively describe the cell cycle as a dynamical system.

Despite the large body of experimental and mathematical works, there are still important aspects of the cell cycle control mechanism that require further studies. For instance, mechanisms that control the cell size increase the survival chance for a cell under different circumstances, e.g., in presence of the molecular noise. However, despite their importance, it is still not clear what underlying control mechanisms maintain the robustness of cell size. Experimental data shows that in most types of living cells, size control is often regulated through feedbacks between the time a cell spends in each phase of cell cycle and the cell volume [17–22]. Nonetheless, the specification of those feedbacks may vary across different living cells, such as fission yeast, mammalian eukaryotes, epithelial cells, and budding yeast.

Since yeasts are genetically tractable in experimental studies, most studies are focused on these unicellular organisms, in particular the budding yeast. Prior studies show that the asymmetric division of budding yeast cells produces smaller daughter cells and larger mother cells. Those small daughter cells spend longer time in G1 phase before they go through the START transition, when a cell makes an irreversible commitment to commence a new cycle. However, those mother cells of the same size commence a new cycle soon after division [23]. This observation suggests that the size control is not strong in mother cells, even if their size is as small as a daughter cell.

Di Talia et al. [23] have studied the effect of molecular noise and size control in variability of the budding yeast cell cycle. More specifically, they used single-cell imaging of fluorescent labeled budding yeast cells to measure the G1 time. According to their observations: 1) the hypothesis that substantial portion of intrinsic noise stems from the noise in gene expression level is confirmed. More specifically, they showed that the variabilities in G1 time decrease with square root of the ploidy and increased dosage of G1 cyclins, 2) size control plays important role in G1 time variability of daughter cells, but not on that of mother cells, and 3) the G1 cyclin genes *CLN2* and *CLN3* dominantly control the regulatory dynamics of the START transition by size control and time control modules. In fact, the size control module enforces longer G1 times to small size daughter cells, but not mother cells. However, the time control module is the same in both daughter and mother cells. The study by Di Talia provides a comprehensive data set that can be used to construct new informative models to quantitatively study the effect of size control module on variability of the cell cycle.

To build such models, deterministic method is the most common approach to study the average properties of protein-protein regulatory network [24–27]. However, this model cannot be generalized to account for the cell-to-cell variabilities observed in experiments. Particularly, analysis of data obtained from single-cell imaging techniques suggests that properties of cell cycle control mechanism involve inevitable intrinsic and extrinsic noise [23, 28]. For instance, partial viability of certain mutant strains reported in [29, 30] is an intrinsically stochastic phenotype caused by substantial variability within the dynamics of cell cycle. Thus, stochastic models are desired to identify the source of variability, quantify the amplitude of noise, and to describe and predict the stochastic phenotypes of mutant strains.

Stochastic models simulate the biochemical reaction network of a living cell using Gillespie’s stochastic simulation algorithm (SSA) [31] to generate discrete time-evolution trajectories of species (genes, mRNAs, and proteins) based on the number of molecules. SSA works accurately if sufficient simulations can be generated. However, SSA is computationally expensive. In fact, the computational complexity of stochastic simulation algorithm scales with the number of reaction firings. Hence, if the dynamical system is large and involves substantial number of reactions with high firing frequency, the computational cost will be extremely high. To reduce this computational complexity, various strategies have been proposed [32–34]. Among the proposed strategies, Haseltine and Rawlings’ (HR) hybrid method is a promising approach.

The HR hybrid method benefits from the multiscale characteristic of biochemical systems. The multiscale feature is inherent in reaction rates and reactant populations inside living cells. For instance, the post-translational reactions (such as phosphorylation/dephosphorylation) during the budding yeast cell cycle are several orders of magnitude more frequent than transcriptional reactions. Moreover, species in a system may also exhibit different scales of populations. For example, mRNAs with average abundance of 5–10 molecules per cell are translated into proteins with average abundance of 1000–10,000 molecules per cell. The HR hybrid method leverages the efficiency of solving ordinary differential equations (ODEs) and accuracy of SSA by integrating both deterministic and stochastic approaches in a single model.

The main contribution of this paper is a new hybrid model that quantitatively describes key characteristics of the cell cycle, such as inter-division times and cell sizes, distribution of mRNAs, as well as the partial viability of specific mutant strains. Building on our previous work in [35], our new model includes the transcripts of the early G1 phase. This feature is in a direct contrast with existing works, such as [27, 35], that disregard the dynamics of early G1 proteins (Cln3 and Bck2) and do not include the G1 cyclin transcripts (*mCln3* and *mBck2*). The proposed model enables studying the effect of noise on G1 cyclins and size control mechanism in the budding yeast cell cycle. In fact, the developed model partitions all 45 proteins and 21 mRNAs, respectively, in fast and slow subsets. Using this partitioning, we obtain the dynamics of the system by solving ODEs for fast subset and applying SSA to slow subset. In addition, we use our model to predict variabilities in mutant strains of G1 phase. Finally, we present comprehensive results for the performance comparison between our proposed model and the experimental observations reported by Di Talia et al. [23].

## Methods and simulation

### Model description

The model we explore in this paper is based on the molecular regulatory network originally proposed by Chen et al. [27]. Chen’s model is a comprehensive deterministic model that accounts for average properties of wild-type budding yeast cells, in addition to the phenotypes of more than 100 mutant strains. Next, we briefly summarize the regulatory network of the Chen’s model.

Cdc28 is the main regulator of the budding yeast cell cycle which is assumed to be constitutively expressed. Cdc28 forms an active kinase by binding to two families of cyclin partners, Cln1-3 and Clb1-6. In the early G1 phase, Cln3 and Bck2 (a backup protein) are the main partners of Cdc28. As a newborn cell grows, the Cln3 and Bck2 proteins accumulate in nucleus to activate the transcription factors SBF and MBF. These two transcription factors are responsible for the production of Cln2 and Clb5. Cln2 accumulation induces the emergence of bud and Clb5 initiates the DNA synthesis. The activation of SBF corresponds to a transition called the START. Once the cell goes through the START transition, it commits to a new cycle. Shortly after the emergence of bud and initiation of DNA synthesis in the S phase, the level of Clb2 rises and the spindle assembly starts to form. Later, the cell goes through another transition called the FINISH, during which a pair of proteins, Cdc20 and Cdh1, become active and facilitate the degradation of Clb2 and Clb5. Through this transition, the level of Clb2 drops, the cell divides and returns to the G1 phase.

Chen’s model describes the protein-protein regulatory network, but does not include the dynamics of mRNAs. There is strong evidence suggesting that the noise in the cell cycle mainly results from the low copy number of mRNAs [23, 29, 36]. Thus, to construct a model that accounts for the variabilities in the cell cycle, it is crucial to incorporate the dynamics of mRNAs along with protein regulators in a stochastic model. Therefore, in our earlier work in [37], we substantially extended Chen’s model by adding the dynamics of 19 important mRNAs. Moreover, we constructed a hybrid stochastic model that not only explains the average properties of the budding yeast cell cycle, it can also reproduce the variability in critical characteristics of the cell cycle, in addition to stochastic phenotypes of specific mutants. Next, we briefly discuss the HR hybrid model.

### The HR hybrid model

Fully stochastic simulation becomes substantially slow when reactants with high abundance or reactions with high frequency are involved [33]. That is because the time complexity of SSA scales with the number of reactions [31]. The HR hybrid method integrates stochastic and deterministic approaches to construct a stochastic model that achieves a good trade-off between accuracy and efficiency. The deterministic method, typically formulated by nonlinear ODEs, is a computationally efficient approach to describe the average properties of a system. Meanwhile, the stochastic counterpart implements the computationally expensive SSA to generate stochastic time-evolution trajectories of the states variables (here the molecule counts of mRNAs). The main idea of the HR hybrid stochastic model is to partition the system of reactions into *fast* and *slow* subsets, apply the computationally expensive SSA only to the slow reactions, and then solve ODEs for the fast subset.

**Fig. 1:**
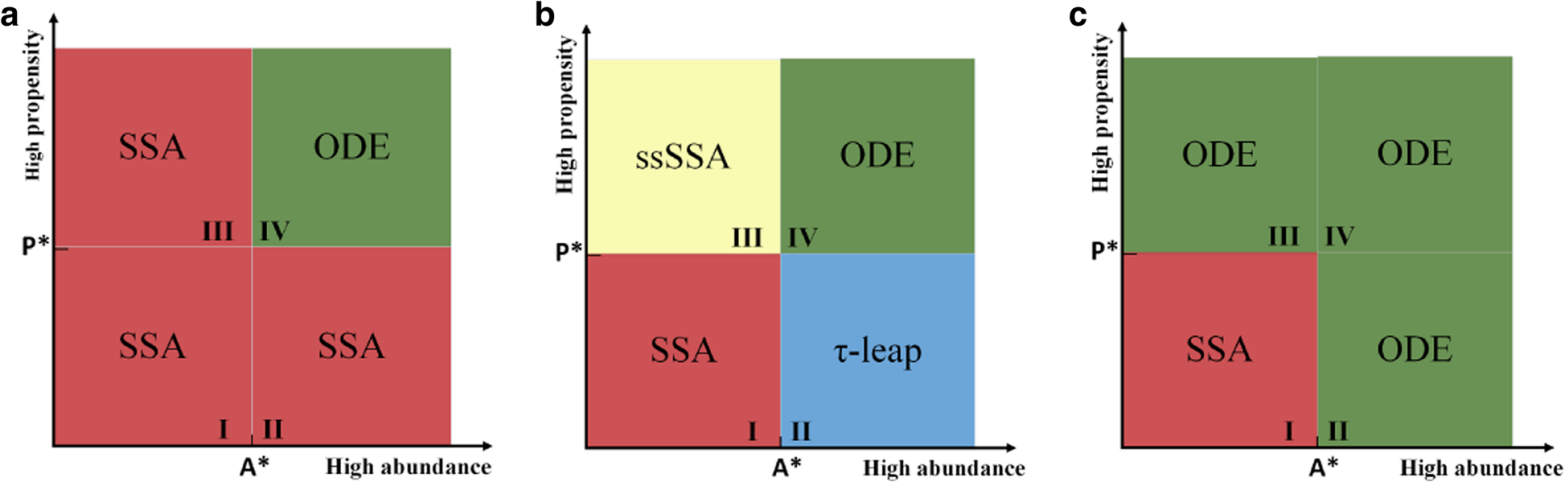
Partitioning strategies for hybrid model. Region I : Slow reactions with low abundance reactant; region II: slow reactions with high abundance reactants; region III: Fast reactions with low abundance reactants; region IV: fast reactions with high abundance reactants. **a** Conservative partitioning strategy by Haseltine and Rawling [38]. **b** Possible partitioning strategy based on the definition of reactions in each region. **c** Partitioning strategy proposed in [41] and used in our model

With predefined thresholds of propensity (P*) and abundance (A*), the reaction channels can be partitioned into four regions as shown in Fig. 1. Region I includes reactants with low abundance and reactions with low propensity. Reactions in the level of gene expression are examples of reaction channels in region I. Due to low copy numbers of species in this region, it is unrealistic to assume that the dynamics of the reactants evolve deterministically over time. For this reason reaction channels in region I are placed in the slow subset where the computationally expensive SSA is applied to accurately simulate the trajectories of state variables. Region IV on contrary, includes reactions with high frequency and reactants with high abundance. Post-translational reactions are examples of the reactions in region IV. Due to the high abundance of the reactants, it is reasonable to approximate the dynamics of state variables in region IV using deterministic methods such as ODEs. Region II and III need more design considerations in order to achieve desired trade-off between accuracy and efficiency.

Different strategies have been proposed in order to choose appropriate simulation methods for reaction channels in region II and III. As described by Fig. 1a, Haseltin and Rawling’s strategy [38] employs SSA for all reactions within regions I, II, and III; and solves ODEs only in region IV. Figure 1b shows a modification to this strategy, which leads to a more efficient model in terms of computational cost. The less conservative strategy in Fig. 1b approximates region II and III, respectively by the *τ*-leap method [39] and the slow scale Stochastic Simulation Algorithm (ssSSA) [40]. The *τ*-leap method leaps over reactions with high-abundance reactants, since inclusion of these reactions may not have a considerable effect on changing the corresponding propensity functions. The ssSSA simulates only slow reactions, assuming that fast reactions are always in partial-equilibrium or steady-state. Figure 1c shows our adopted partitioning strategy that simulates all reactions in region I (slow subset) by SSA, and reactions in all other regions (fast subset) by ODEs. In Liu et al. [41], this approach was applied to a three-variable model of cell cycle and was shown to achieve much higher efficiency, while maintaining very good accuracy in comparison with other conservative partitioning strategies as well as a full stochastic model.

In our model the reaction channels in region I includes the synthesis and degradation of transcripts. After such partitioning we apply the HR hybrid algorithm to simulate the trajectories of state variables. The step by step algorithm is provided in [35, 37] and more implementation details can be found in [42]. In those works, we have shown that placing the dynamics of mRNAs into SSA regime and solving ODEs for protein regulatory network leads to sufficiently accurate results, and significantly reduces the computational cost.

**Fig. 2:**
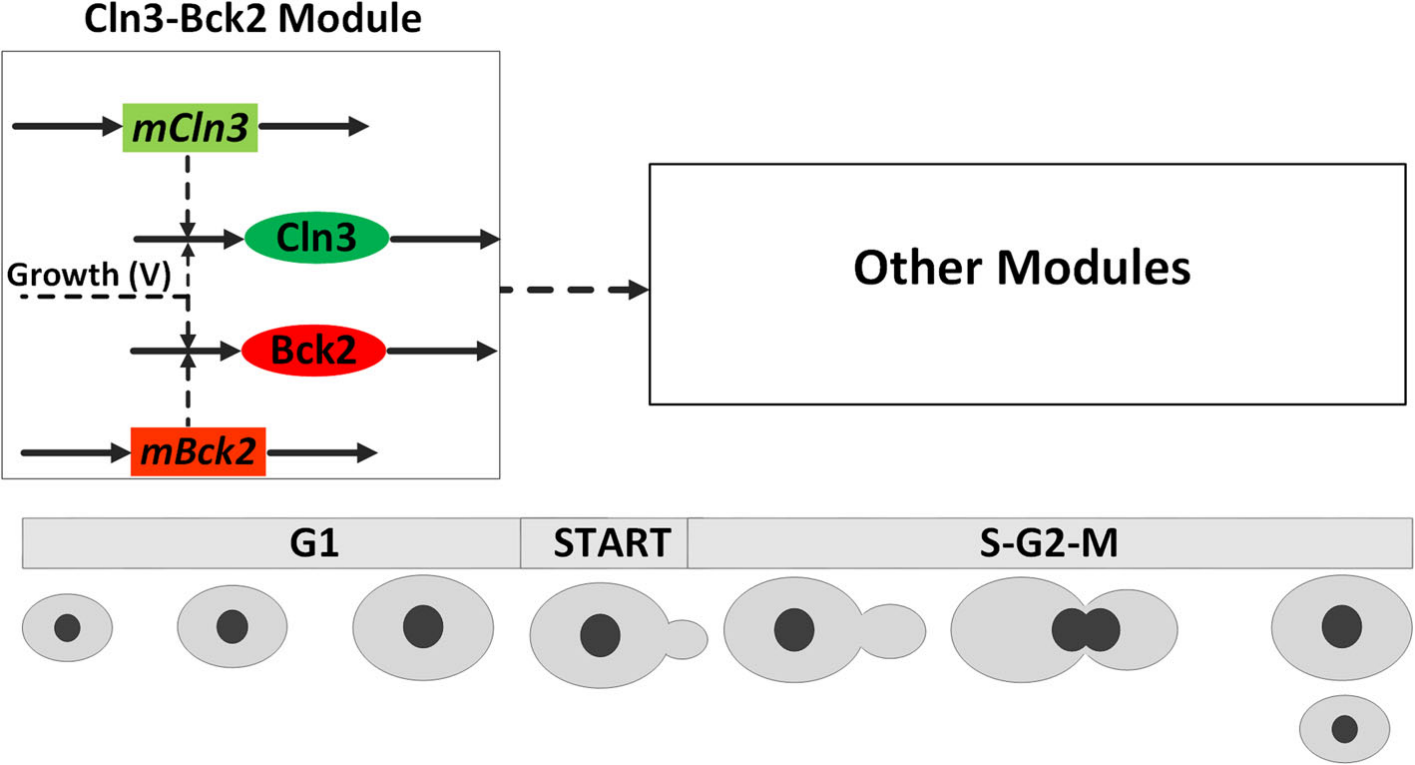
Modified Cln3-Bck2 module for budding yeast cell cycle model. Solid arrows represent synthesis/degradation reactions and dashed arrows represent activation. The *mCln3* and *mBck2* are unregulated mRNAs constitutively transcribed. As cell grows in G1 phase, Cln3 and Bck2 proteins accumulate and the cell goes through the START transition, during which the cell commits to a new round of division. The START transition is identified by the activation of two important transcription factors (SBF and MBF), which are included in *Other Modules* and not presented here
  2 $$\begin{array}{*{20}l} [\text{\!Bck2}] &= \text{[mass]}\cdot B_{0} \end{array} $$
  Here *C*_0_ determines the maximum activity of Cln3, *D*_n3_ is the dosage of *CLN3* gene, *J*_n3_ is the Michaelis Menton constant, and *B*_0_ is the Bck2 constant. We note that [*X*] denotes the concentration of species *X*. These algebraic equations present an underlying assumption that the aforementioned proteins are always in steady states. We relax this assumption and modify these two algebraic equations into corresponding ODEs in (3) and (4):
  3 $$\begin{array}{*{20}l} \frac{\text{dCln3}}{\text{d}t} &= k_{\text{s,n3}}\cdot V^{2}\cdot \text{mCln3} -(k_{\text{d,n3}} - k_{\text{g}})\cdot \text{Cln3}, \end{array} $$
  4 $$\begin{array}{*{20}l} \frac{\text{dBck2}}{\text{d}t} &= k_{\text{s,k2}}\cdot V\cdot \text{mBck2} -(k_{\text{d,k2}}- k_{\text{g}})\cdot \text{Bck2}, \end{array} $$
  where *k*_g_ is the growth rate of the cell, *V* indicates the volume of the cell, *k*_s,n3_ and *k*_d,n3_ are the synthesis and degradation rates of Cln3 and *k*_s,k2_ and *k*_d,k2_ are the synthesis and degradation rates of Bck2. The growth rate is defined as *k*_g_=ln2/MDT, where MDT is the mass doubling time of the culture. MDT is measured experimentally for different nutrient cultures. For instance, the MDT for glucose is observed to be approximately 90 minutes and thus, the growth rate of glucose culture is estimated as ln2/90≈0.0077 min^−1^. The synthesis and degradation rates are estimated as follow: we first estimate the degradation rate defined by *k*_d,p_=ln2/*τ*_p_, where *τ*_p_ is the half-life time of the protein P. Then, we estimate the synthesis rate such that the average abundance of the protein matches with experimental observation reported in [15]. Table 1 lists the estimated parameters.

In this paper, we substantially improve our extended model in [37] as follows:

- First, we incorporate the dynamics of two early G1-phase proteins, Cln3 and Bck2 as depicted in Fig. 2. In fact, Cln3 and Bck2 play important roles in the Start transition and thus are necessary to be included into the model. In Chen’s original model [27] the activities of Cln3 and Bck2 were formulated by algebraic Eqs. (1) and (2), respectively.
  1 $$\begin{array}{*{20}l} [\text{\!Cln3}] &=\frac{C_{0}\cdot D_{\text{n3}}\cdot\text{[mass]}}{(J_{\text{n3}} + D_{\text{n3}}\cdot\text{[mass]})}, \end{array} $$

**Table 1:** Estimated parameters (min^−1^) in fast subset of the extended model

Parameter	Value	Parameter	Value
*k*_s,n3_	0.0015	*k*_d,n3_	0.12
*k*_s,k2_	0.024	*k*_d,k2_	0.14
*k*_g_	0.0072
  The reason we modified the algebraic equations in (1) and (2) into ODEs in (3) and (4) is to follow the experimental observations in [28, 43]. These observations show that *CLN3* gene is down-regulated in a new born daughter cell. More specifically, Di Talia et al. [28] observed that the *CLN3* gene is about 3 times less expressed in daughter cells in comparison with a newly divided mother cell. This can be modeled by choosing a partitioning ratio of 25:75 for the distribution of Cln3 and Bck2 between daughter and mother cells at division. To apply this partitioning ratio, the dynamics of Cln3 and Bck2 should be formulated by ODEs rather than algebraic equations, since this particular partition rule can be only applied to state variables, not those species in algebraic equations. That is because the abundance of species formulated by algebraic equations completely depends on other species and thus, they cannot be partitioned with individual rules.
- The second modification is that we coupled the synthesis rate of Cln3 quadratically to growth as (*V*^2^). The reason is that to have the abundance of Cln3 to be indicative of cell growth, this protein must be synthesized in an accelerated rate in comparison with other proteins such as Cln2 or Clb5 [44]. Through many different sorts of experimental measurements [16, 45–47], it has been observed that the size control at Start transition is primarily through the action of Cln3 proteins. For instance, it has been shown that the translation rate of *mCln3* with low intrinsic initiation rate, enhances more significantly by increasing the availability of ribosomal precursors [47]. Moreover, the translation of *mCln3* is affected by specific sequence that is sensitive to cell growth rate [45]. Additionally, the *mCln3* level rises more than other mRNAs such as *mCln2* in the presence of glucose [46]. These observations support the idea that Cln3 has stronger dependencies with cell size in comparison with other proteins such as Cln2. For this reason we believe that a quadratic coupling of size and Cln3 better reflects the significant role of Cln3 in Start transition. We note that *mass* is an indicator of the cell size in Chen’s model. We also alter this variable *mass* by replacing it with a volume variable (*V*), which is a more sensible metric of size and can be directly compared with experimental data.
- The last modification includes the dynamics of early G1 transcripts in the subset of slow reactions. Table 2 lists the synthesis and degradation rates of the transcripts, *mCln3* and *mBck2*. To estimate the corresponding reaction rates, the degradation rate is defined first. For an mRNA denoted by *m*, the degradation rate is *k*_dm_=ln2/*τ*_m_, where *τ*_m_ is the half-life time of transcript *m*. In our model, the half-life time values are in the range of 5–10 minutes, consistent with the experimental measurements of Miller et al. reported in [14]. The synthesis rate, *k*_sm_, is also estimated such that the average abundance of the mRNA is consistent with experimental measurements: 〈*m*〉=*k*_sm_/*k*_dm_, where 〈*m*〉 denotes the average abundance of the corresponding mRNA, *m*.

**Table 2:** Modified reactions and propensities in slow subset of the extended model

Reaction	Reaction rate	Parameter value	Propensity
*ϕ*→mCln3	*k*_s,mcln3_	0.5	*k*_s,mcln3_·*V*
mCln3→*ϕ*	*k*_d,mcln3_	0.10	*k*_d,mcln3_·*mCln*3
*ϕ*→mBck2	*k*_s,mbck2_	0.7	*k*_s,mbck2_·*V*
mBck2→*ϕ*	*k*_d,mbck2_	0.14	*k*_d,mBck2_·*mbck*2

We apply the HR hybrid stochastic algorithm [37] to the modified model with the listed parameters in Tables 1 and 2.

## Results and discussion

**Fig. 3:**
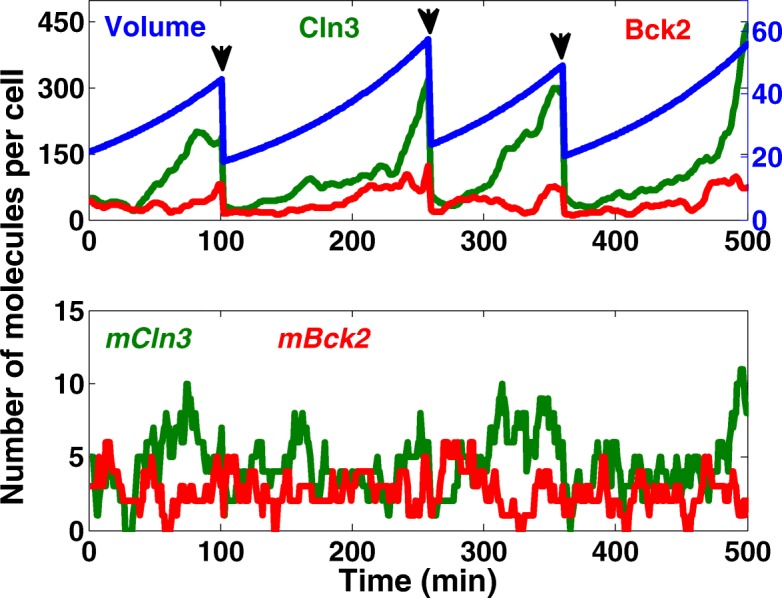
Oscillatory dynamics of proteins and mRNAs in the Cln3-Bck2 module. The temporal dynamics of specific proteins (top panel) and mRNAs (bottom panel) generated by an independent run of the hybrid stochastic model is demonstrated base on numbers of molecules per cell. The volume of cell (solid blue line) grows exponentially and divides asymmetrically (the division is indicated by arrows) between daughter and mother cells. This asymmetric division follows the experimental observations by Di Talia et al. [23, 28]. Except for Cln3 that divides with a ratio of 25:75 between daughter and mother, the rest of proteins along with the volume are partitioned with a ratio of 40:60. In simulation this is implemented by setting the initial values of the progenies for the next simulation run according to the aforementioned ratios

The modified hybrid stochastic model is used to generate sufficiently large populations of daughter and mother cells (at least 5,000 cells in each independent simulation run) starting from one cell at *t* = 0. Figure 3 shows the oscillatory dynamics of the protein and mRNA molecules in the modified Cln3-Bck2 module. The cell volume grows exponentially with time and divides asymmetrically between the daughter and mother cells. As the cell grows in size, the levels of Cln3 and Bck2 proteins rise. The average abundance of the proteins and mRNAs are computed from the simulation results. The Cln3 protein, with an average abundance of 186 molecules per cell, matches very well with experimental observations (216 molecule per cell) [15]. The unregulated mRNAs, *mCln3* and *mBck2*, are constitutively expressed (see Fig. 3 bottom panel) with an average abundance of about 5, which is within the experimental range of 5-10 copies of unique mRNAs per cell. First, we show that gene expression noise introduces significant variability to cell cycle. One way to look into this is through increasing the ploidy, because ploidy increases the average number of transcripts. Thus, if the source of noise stems from transcripts, the ploidy will reduce the variability. Experimental studies show that when the ploidy level is doubled, the abundance of all cellular components as well as the volume of the cell are doubled while the concentration of species remains the same. In our simulations, in order to generate populations of diploid and tetraploid wild-types, the synthesis rates of all transcripts are estimated such that the average number of transcripts are, respectively, twice and four times larger than haploid wild-type cells. Moreover, the average volume of the cell is increased accordingly. To quantify the variability the coefficient of variation (CV = standard deviation/mean) is computed for each population.

**Fig. 4:**
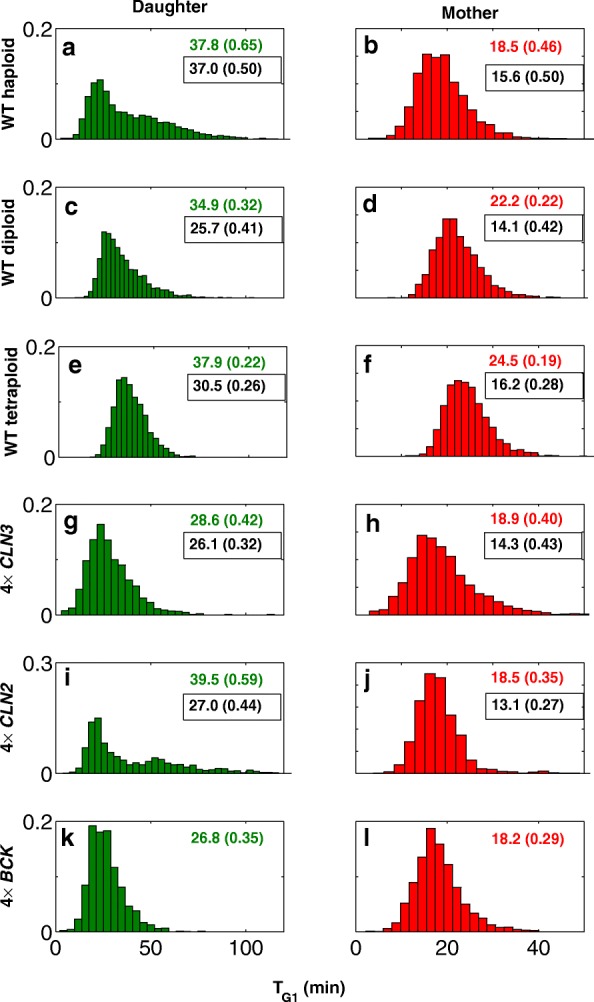
Increasing the ploidy or the G1 cyclin transcripts decreases the variabilities in G1 time. The histograms of G1 time duration for daughter and mother cells are generated by simulation of sufficiently large population of cells in different simulation scenarios. The summary statistics is given for quantitative comparison. Mean (CV) of simulation is compared with experimental data (in box). **a**, **b**: WT haploid; **b**, **c**: WT Diploids; **e**, **f**: WT tetraploid, **g**, **h**: 4 ×*CLN3*; **i**, **j**: 4 ×*CLN2*; **k**, **l**: 4 ×*Bck2*; left panels: daughters; right panels: mothers

Figure 4a-l shows histogram of the G1 time, generated from simulations of sufficiently large populations of haploids, diploids, and tetraploids for both daughter and mother cells. Comparing the covariances (CVs) of daughter cells (on left) and mother cells (on right), we notice that the G1 variability is reduced from top to bottom in both daughter and mother cells. Thus, we can infer that the noise in gene expression level induces substantial variability to the cell cycle system.

Next, we investigate the effect of the noise introduced by G1 cyclin genes *CLN3* and *CLN2* along with *BCK2* by increasing the dosage of the corresponding transcripts. To this end, in simulations, the synthesis rates of the transcripts are estimated such that their average abundance is enlarged by 4 folds. Figure 4g-l demonstrates the histogram of the G1 time for cell population with increased dosage of genes. Decreasing pattern of CVs in comparison with haploid wild-type cells is evident for both daughter and mother cells. Thus, the results of our model is consistent with the hypothesis that the low copy number of transcripts result in substantial variability in cell cycle. In particular, in the G1 phase, this variability is more considerable due to limited number of species.

This analysis shows how the noise reduction in level of gene expression results in reduction in variability of the G1 time. Next, we investigate the effect of size control on such variability. Deterministic size control has long been proposed as a mechanism that regulates the G1 time [17, 19]. This means that cells stay in G1 phase until they reach a critical size. Such deterministic size control ensures that all cells bud at the same size. Since the cell size at birth is variable, the size control would assure smaller cells stay long enough to reach the critical size. This introduces variability in G1 duration. To quantify such variability in experiment, instead of geometric volume estimation which is less reliable, Di Talia et al. related size at bud, *V*_bud_, to size at birth, *V*_birth_, through the amount of time the cell spends in G1 phase (*T*_*G*1_) by:

5 $$\begin{array}{*{20}l}  V_{\text{bud}} = V_{\text{birth}}\mathrm{e}^{k_{\text{g }}T_{\text{G1}}}, \end{array} $$

where *k*_g_ is the growth rate of the media. We notice that the underlying assumption of (5) is that the population growth is exponential, which is consistent with experimental observations for the budding yeast [23]. Equation (5) leads to *k*_g_*T*_G1_=ln(*V*_bud_)−ln(*V*_birth_), where *k*_g_*T*_G1_ can be used as an indicator of the volume change during the G1 phase. In simulations, however, change in volume of cells from birth to bud can be directly recorded by defining specific flags. Therefore, we use the volume change in our simulations instead of *k*_g_*T*_G1_.

**Fig. 5:**
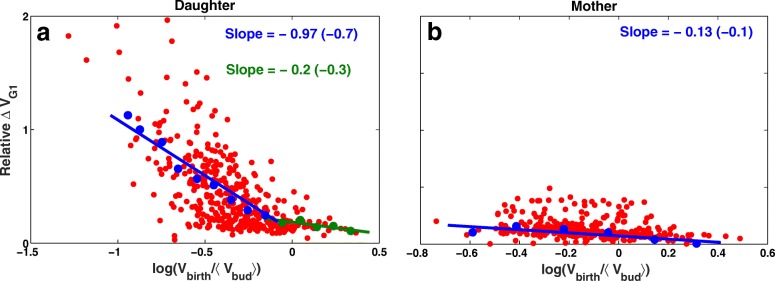
Cell cycle variability and size control for both daughter and mother cell. Size control leads to a negative correlation between cell size at birth and growth in G1 phase. Plotting the logarithm of size at birth, *V*_birth_, scaled with the average volume at bud, 〈*V*_bud_〉, with respect to relative growth in G1 ((*V*_bud_−*V*_birth_)/*V*_birth_) quantifies how strong the G1 size control is in daughter (**a**) and mother cells (**b**). A slope of −1 would indicate a perfect size control, whereas a slope of 0 indicates a lack of size control. Wild-type budding yeast shows imperfect size control in glucose

Figure 5a and b shows the correlation between the relative change in volume during G1 phase and the scaled volume at birth for mother and daughter cells in glucose. The growth of mother cells in G1 phase is almost independent of the size at birth. There is a very good match between the experimental observations (slope =−0.1) and our model (slope =−0.13). For daughter cells, however, strong size control is evident (see Fig. 5b). Small daughter cells need to stay longer and grow larger in G1 phase before they go through START transition and commit to a new cycle. The size control is stronger in smaller daughters (slope =−0.97) compared with the larger ones (slope =−0.2). The quantified correlations are, respectively, comparable with (slope =−0.7) and (slope =−0.3) from experiments [23].

**Fig. 6:**
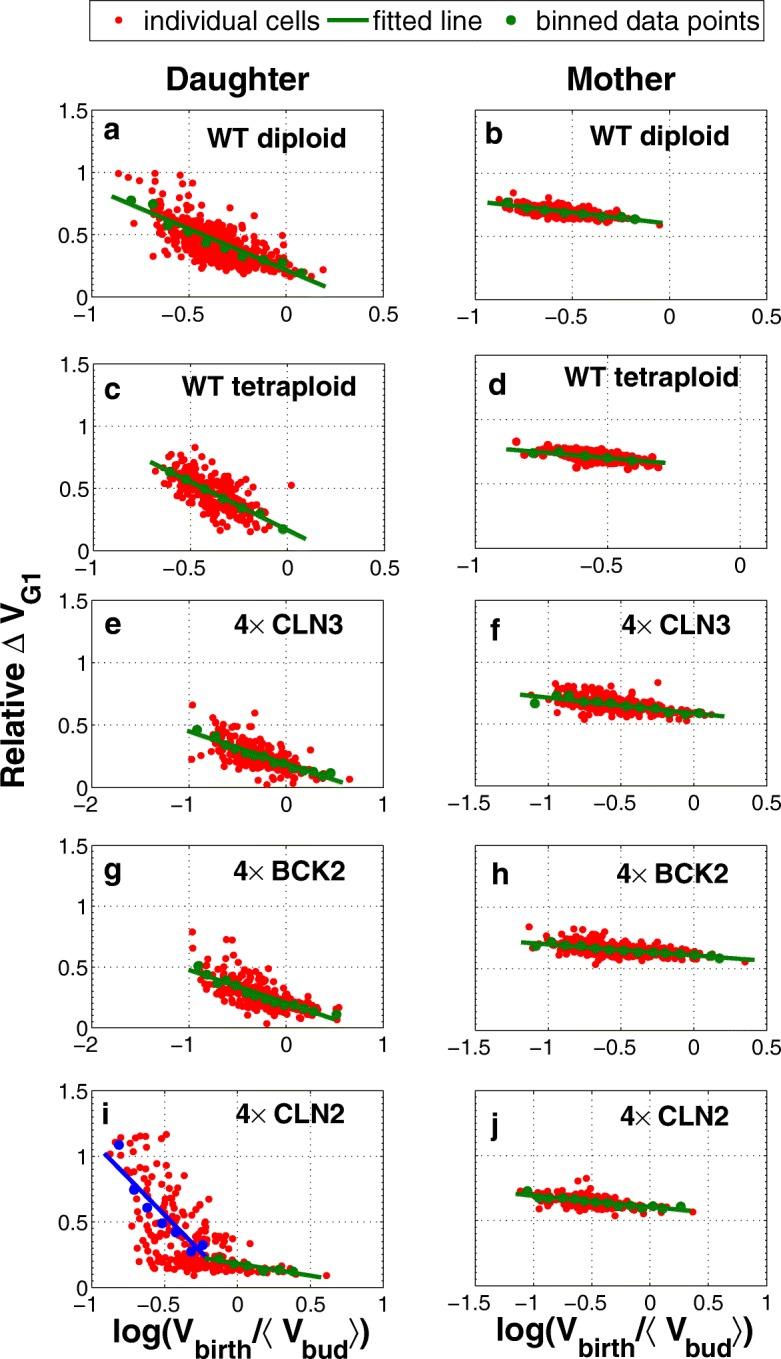
Size control variability is reduced by ploidy and increasing the copy numbers of G1 cyclins in both mother and daughter cells. The correlation between the relative change in volume of a cell during the G1 phase (Relative*ΔV*_G1_) and the normalized logarithm of cell size at birth (log(*V*_birth_/〈*V*_bud_〉) is shown for ploidy (**a-d**) and cells with increased dosage of genes (**e-j**). The quantified correlation shows that the cell size independent noise is decreased by ploidy and also by increasing the copy numbers of G1 cyclins as well as BCK2

This analysis quantitatively describes two causes for G1 variability: 1) variability that is introduced by size control and 2) variability that is independent of size and stems from molecular noise [23]. The simulation results of cells with increased copies of genes provides more quantitative evidence in this regards. The efficient size control observed in wild-type daughters (see Fig. 5a) is reduced by ploidy and increasing the dosage of G1 cyclins. Figure 6 shows the correlation between relative volume growth during G1 phase and the scaled volume at birth. The linear fit to the binned data demonstrates a smaller slop in diploid and tetraploid daughters in comparison with haploid wild-type in Fig. 5a. Similarly, the less efficient size control is evident in daughter cells with increased dosage of *CLN3*, *BCK2*, and *CLN2*. In addition, Fig 6. e, g, and i shows that increasing the number of *CLN3* and *BCK2* copies substantially alters the size control while the increased dosage of *CLN2* does not. The lack of size control in mother cells is also evident in right-hand side panels in Fig. 6. Our model describes the stochastic size control of the budding yeast cell cycle very well and is quantitatively consistent with experimental observations by Di Talia et al. [23]

Thus far, using different analysis we have shown that our hybrid stochastic model captures many properties of the cell cycle and matches very well with experimental data. Next we, use the proposed model to predict the variability in phenotypes of mutant strains in START network, more specifically the genes that play role in size control. The average phenotype of mutants have been modeled deterministically first by Chen et al. in [26, 27] and recently by Kraikivski et al. in [48]. However, the variability of cell cycle properties in different mutant strains needs stochastic modeling and yet is not addressed by prior works.

**Fig. 7:**
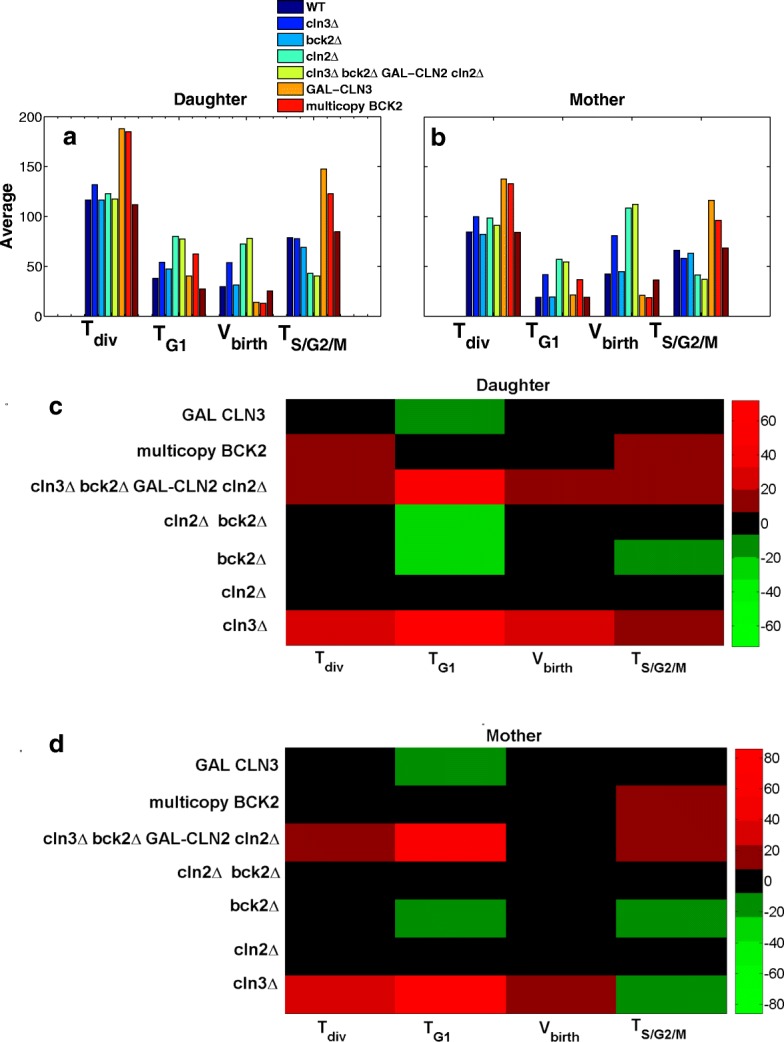
Mutant phenotypes of selected strains in START network. The average (**a** and **b**) and CV (**c** and **d**) of critical properties of cell cycle (including cycle time (*T*_div_), G1 time *T*_G1_, duration of S/G2/M phase, (*T*_S/G2/M_) and size at birth (*V*_birth_) are computed for simulated population of selected mutant strains for both daughter and mother cell. Time is in min and volume in fL

Figure 7a and b shows the results from hybrid stochastic simulation for some of viable mutants of START network. Important properties of the cell cycle including division time (*T*_div_), G1 time (*T*_G1_), volume at birth (*V*_birth_), and S/G2/M duration (*T*_S/G2/M_) are computed for the population of the mutants. According to results in Fig. 7a and b for both daughter and mother cells, the average properties of the cell cycle predicted by our proposed model is consistent with the experimental observations reported in [27]. For instant, for those strains with deleted G1 cyclin genes such as *cln*3*Δ* and *cln*2*Δ*, the G1 time is significantly longer than the wild-type cell. That is because the budding is delayed due to deletion of these genes. For a similar reason, the G1 phase is prolonged in *bck*2*Δ* and *cln*2*Δbck*2*Δ*. Moreover, since these strains stay longer in G1 phase, they grow larger before they commence a new cycle. For this reason, the size of these mutants after division (at birth) is larger on average in comparison with wild type cell [26, 27]. *GAL*−*CLN*3 and multicopy-*BCK*2 with increased activity of *CLN3* and *BCK2* produce cells with smaller size. Such phenotype of these mutant strains is precisely described by our model.

Figure 7c and d shows the percent change in variability of cell cycle properties under perturbation of *CLN3*, *CLN2*, and *BCK2* genes compared with wild-type cell. According to the prediction of the model, deletion of *CLN3* gene induces highest amount of noise into almost all properties of the cell cycle, particularly to the G1 phase. The G1 time is highly variable because Cln3 protein is the cyclin that contributes the most in G1 phase (by activation of SBF which is the transcription factor of Cln2). Hence, in the absence of *CLN3* gene, the START transition becomes sloppy. Another observation is that *cln3* deletion induces more variability to the mother cell than the daughter cell. This can be explained by the asymmetric division of Cln3 and *mCln3* in budding yeast. Considering the experimental observations in [28], we partition both Cln3 and *mCln3* with ratio of 25:75 between daughter and mother cells. Thus, the absence of Cln3 protein has more considerable effect in variability of mother cells.

Bck2 is a back-up protein in G1 phase. In absence of this protein, Cln3 is able to drive the cell to START transition and hence, deletion of the *bck2* does not greatly affect the properties of cell cycle. However, increasing the dosage of this gene in multicopy-*BCK*2 significantly decreases the variability of G1 phase. Clearly, that is because multiple copies of the gene decrease the fluctuation in level of gene expression, and consequently, in G1 duration.

Deleting *CLN2* gene in *cln*2*Δ* strain does not considerably change the variability in cell size or other phases of the cell cycle, except for G1 phase of daughter cell. This phenotype is not consistent with our expectation and under current parameterization, this observation requires more investigation. *CLN1* and *CLN2* genes have overlapping functions in formation of bud and spindle pole body duplication [26]. Due to the similarity of Cln1 and Cln2 proteins, the combined activity of these proteins is typically presented by only Cln2 to simplify the model. One possible solution to study the dynamics of this family of cyclins in more detail is to include the dynamics of Cln1 and *mCln1* in the model.

Experimental studies show that the noisiest phase of the budding yeast cell cycle is G1 with coefficient of variation equal to 50% for both daughter and mother cells. Thus, one may infer that the variability in other phases (S/G2/M) is dictated by the G1 variability. However, Debashis et al. in [30] showed that the variability in cell cycle times and volume at birth are driven by the variability of the S/G2/M phase (bud phase), rather than the G1 (unbud) phase.

**Fig. 8:**
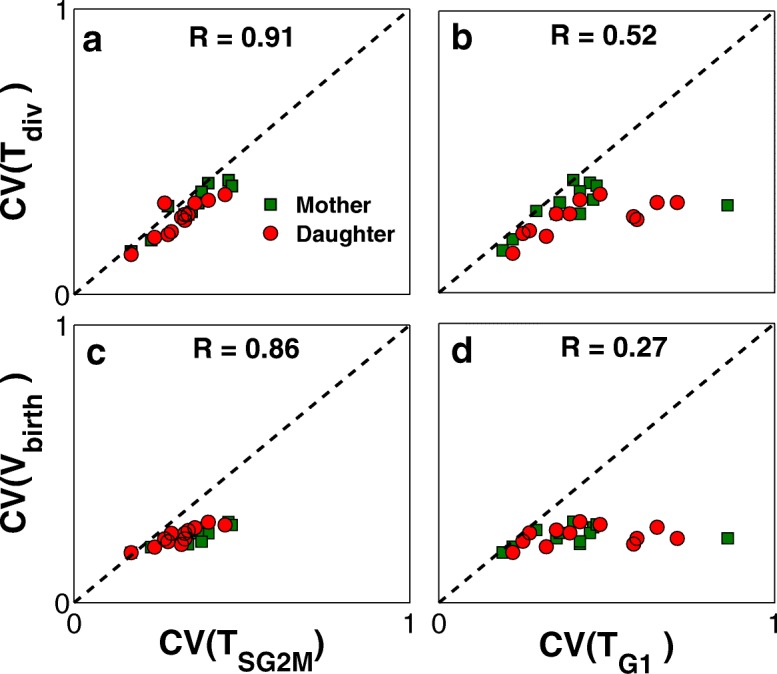
Correlation between the Variability of size and duration of different phases of cell cycle. CVs of *T*_div_ (**a** and **b**) and *V*_birth_ (**c** and **d**) are correlated with CVs of *T*_S/G2/M_ and *T*_G1_ for simulated population of haploids, diploids, tetraploids as well as several viable mutant strains. Correlation coefficients (R) are computed and given on each panel

Next, we study the correlation between variability in division time and size at birth with respect to variability in duration of G1 and S/G2/M phases. To this end, the CVs of G1, S/G2/M, and division time along with the cell volume at birth are computed from simulation of the following populations: wild-type haploids, diploids and tetraploids in glucose, mutant strains presented in Fig. 7, and the cells with increased dosage of genes presented in Fig. 8. Moreover, the product-moment correlation coefficient is computed to quantify such correlations.

Figure 8a-d demonstrates that variability in cycle time and volume with respect to variability in duration of G1 and S/G2/M phases. There is strong correlation (R = 0.91) between the CVs of division time and S/G2/M duration (see Fig. 8a), while such correlation is less evident for G1 duration with coefficient of correlation equal to 0.52 (see Fig. 8b). Similarly, Fig. 8c and d shows that CVs of birth size are more strongly correlated to less noisy phase of the cell cycle (S/G2/M), rather than the G1 phase. The reason for such non-intuitive observation lies in size control mechanism. According to the results in Fig. 5, size control imposes a negative correlation between the size at birth and G1 duration in order to make the cells bud at the same size such that the variability in size is minimized before START transition [30]. Hence, major variability of the cell size from birth to division takes place after START and during the S/G2/M phase.

## Conclusion

In this paper, we present a new hybrid stochastic (ODE/SSA) model to address the variability in the budding yeast cell cycle introduced by noise in level of gene expression and stochastic size control. We have significantly modified existing works by adding a modified Cln3-Bck2 module. This module is required to study the G1 variability due to importance of Cln3 and Bck2 proteins at the early G1 phase. We have applied the HR hybrid method in our developed model to maintain a good trade-off between accuracy and efficiency by integrating deterministic and stochastic simulations. We note that the full stochastic simulation is computationally expensive to be used in our large model that includes 66 proteins and mRNAs. In order to incorporate the inherent molecular fluctuations of the cell cycle in our model, we have enhanced the protein-protein regulatory module of the Chen’s deterministic model to a gene-protein regulatory network, where *mCln3* and *mBck2* are incorporated.

We validate our model though comprehensive numerical simulations and present several comparisons with wet-lab experimental data. With manageable computational complexity, our approach has successfully accounted for a broad range of single-cell experimental observations on wild-type and mutant cells. In particular, our model has confirmed that substantial amount of deleterious noise in cell cycle stems from the low copy number of mRNAs in early G1 phase. The imperfect sizer of the budding yeast control mechanism has also been predicted by the proposed model. Moreover, the effect of size control on variability of certain mutant phenotypes in Cln3-Bck2 module have been quantitatively described. The proposed model has yielded promising preliminary results that can be used to build more comprehensive models of size control regulatory network.

## Abbreviations

CV: Coefficient of variation
HR: Haseltine and Rawlings
MDT: Mass doubling time
ODEs: Ordinary differential equations
SSA: Stochastic simulation algorithm
ssSSA: Slow scale Stochastic Simulation Algorithm

## References

[1] Hartwell LH, Unger MW (1977). Unequal division in saccharomyces cerevisiae and its implications for the control of cell division. J Cell Biol.

[2] Carter BLA, John PCL (1981). The control of cell division in saccharomyces cerevisiae. The Cell Cycle.

[3] Murray A, Hunt T (1993). The cell cycle: An introduction.

[4] Alberts B, Bray D, Lewis J, Raff M, Roberts K, Watson JD (1994). Molecular biology of the cell, 3rd ed.

[5] Nasmyth K (1995). Evolution of the cell cycle. Philos Trans R Soc Lond B.

[6] Nasmyth K (1996). At the heart of the budding yeast cell cycle. Trends Genet.

[7] Botchan M (1996). Coordinating dna replication with cell division: current status of the licensing concept.. Proc Natl Acad Sci USA.

[8] Tyson JJ, Novak B, Odell GM, Chen KC, Thron CD (1996). Chemical kinetic theory as a tool for understanding the regulation of m-phase promoting factor in the cell cycle. Trends Biochem Sci.

[9] Amon A (1997). Regulation of b-type cyclin proteolysis by cdc28-associated kinases in budding yeast. EMBO J.

[10] Biggins S, Murray AW (1998). Sister chromatid cohesion in mitosis. Curr Opin Cell Biol.

[11] Leatherwood J (1998). Emerging mechanisms of eukaryotic dna replication initiation. Curr Opin Cell Biol.

[12] Zachariae W, Schwab M, Nasmyth K, Seufert W (1998). Control of cyclin ubiquitination by cdk-regulated binding of hct1 to the anaphase promoting complex. Science.

[13] Miller ME, Cross FR (2001). Mechanisms controlling subcellular localization of the g1 cyclins cln2p and cln3p in budding yeast. Mol Cell Biol.

[14] Miller C., Schwalb B., Maier K., Schulz D., Dumcke S., Zacher B., Mayer A., Sydow J., Marcinowski L., Dolken L., Martin D. E., Tresch A., Cramer P. (2014). Dynamic transcriptome analysis measures rates of mRNA synthesis and decay in yeast. Molecular Systems Biology.

[15] Ghaemmaghami S, Huh W-K, Bower K, Howson RW, Belle A, Dephoure N, O’Shea EK, Weissman JS (2003). Global analysis of protein expression in yeast. Nature.

[16] Lord PG, Wheals AE (1980). Asymmetrical division of saccharomyces cerevisiae. J Bacteriol.

[17] Johnston GC, Pringle JR, Hartwell LH (1997). Coordination of growth with cell division in the yeast saccharomyces cerevisiae. Exp Cell Res.

[18] Goranov AI, Cook M, Ricicova M, Ben-Ari G, Gonzalez C, Hansen C, Tyers M, Amon A (2009). The rate of cell growth is governed by cell cycle stage. Genes Dev.

[19] Ferrezuelo F, Colomina N, Palmisano A, Gari E, Gallego C, Csikasz-Nagy A, Aldea M (2012). The critical size is set at a single-cell level by growth rate to attain homeostasis and adaptation. Nat Commun.

[20] Schmoller KM, Turner JJ, Koivomagi M, Skotheim JM (2015). Dilution of the cell cycle inhibitor whi5 controls budding-yeast cell size. Nature.

[21] Soifer I, Robert L, Amir A (2016). Single-cell analysis of growth in budding yeast and bacteria reveals a common size regulation strategy. Curr Biol.

[22] Turner JJ, Ewald JC, Skotheim JM (2012). Cell size control in yeast. Curr Biol.

[23] Di Talia S, Skotheim JM, Bean JM, Siggia ED, Cross FR (2007). The effects of molecular noise and size control on variablility in the budding yeast cell cycle. Nature.

[24] Tyson JJ (1991). Modeling the cell division cycle: Cdc2 and cyclin interactions. PNAS.

[25] Tyson JJ, Novak B (2001). Regulation of the eukaryotic cell cycle: molecular antagonism, hysteresis, and irreversible transitions. J Theor Biol.

[26] Chen KC, Csikasz-Nagy A, Gyorffy B, Val J, Novak B, Tyson JJ (2000). Kinetic analysis of a molecular model of the budding yeast cell cycle. Mol Biol Cell.

[27] Chen KC, Calzone L, Csikasz-Nagy A, Cross FR, Novak B, Tyson JJ (2004). Integrative analysis of cell cycle control in budding yeast. Mol Biol Cell.

[28] Di Talia S, Wang H, Skotheim JM, Rosebrock AP, Futcher B, Cross FR (2009). Daughter-specific transcription factors regulate cell size control in budding yeast. PLoS Biol.

[29] Ball DA, Ahn T, Wang P, Chen KC, Cao Y, Tyson JJ, Peccoud J, Baumann WT (2011). Stochastic exit from mitosis in dudding yeast. Cell Cycle.

[30] Barik D, Ball DA, Peccoud J, Tyson JJ (2016). A stochastic model of the yeast cell cycle reveals roles for feedback regulation in limiting cellular variability. PLoS Comput Biol.

[31] Gillespie DT (1976). A general method for numerically simulating the stochastic time evolution of coupled chemical reactions. J Contemp Math.

[32] Gillespie DT, Petzold LR (2003). Improved leap-size selection for accelerated stochastic simulation. J Chem Phys.

[33] Gibson MA, Bruck J (2000). Efficient exact stochastic simulation of chemical systems with many species and many channels. J Chem Phys.

[34] Cao Y1, Li H, Petzold L (2001). Efficient formulation of the stochastic simulation algorithm for chemically reacting systems. J Chem Phys.

[35] Wang S, Ahmadian M, Chen M, Tyson JJ, Cao Y (2016). A hybrid stochastic model of the budding yeast cell cycle control mechanism. Proceedings of the 7th ACM International Conference on Bioinformatics, Computational Biology, and Health Informatics.

[36] Ball DA, Adames NR, Reischmann N, Barik D, Franck CT, Tyson JJ (2013). Measurement and modeling of transcriptional noise in the cell cycle regulatory network. Cell Cycle.

[37] Ahmadian M, Wang S, Tyson JJ, Cao Y (2017). Hybrid ode/ssa model of the budding yeast cell cycle control mechanism with mutant case study. Proceedings of the 8th ACM International Conference on Bioinformatics, Computational Biology, and Health Informatics.

[38] Haseltine EL, Rawlings JB (2002). Approximate simulation of coupled fast and slow reactions for stochastic chemical kinetics. J Chem Phys.

[39] Gillespie DT (2001). Approximate accelerated stochastic simulation of chemically reacting systems. J Chem Phys.

[40] Cao Y, Gillespie DT, Petzold LR (2005). The slow-scale stochastic simulation algorithm. J Chem Phys.

[41] Liu Z, Pu Y, Li F, Shaffer CA, Hoops S, Tyson JJ, Cao Y (2012). Hybrid modeling and simulation of stochastic effects on progression through the eukaryotic cell cycle. J Chem Phys.

[42] Wang S, Chen M, Watson LT, Cao Y (2017). Efficient implementation of the hybrid method for stochastic simulation of biochemical systems. J Micromech Mol Phys.

[43] Laabs TL, Markwardt DD, Slattery MG, Newcomb LL, Stillman DJ, Heideman W (2003). Ace2 is required for daughter cell-specific g1 delay in saccharomyces cerevisiae. Proc Natl Acad Sci U S A.

[44] Barik D, Baumann WT, Paul MR, Novak B, Tyson JJ (2010). A model of yeast cell-cycle regulation based on multisite phosphorylation. Mol Syst Biol.

[45] Polymenis M, Schmidt EV (1997). Coupling cell division to cell growth by translational control of the g1 cyclin cln3 in yeast. Genes Dev.

[46] Newcomb LL, Hall DD, Heideman W (2002). Azf1 is a glucose-dependent positive regulator of cln3 transcription in saccharomyces cerevisiae. Mol Cell Biol.

[47] Lodish HF (1974). Model for the regulation of mrna translation applied to haemoglobin synthesis. Nature.

[48] Kraikivski P, Chen KC, Laomettachit T, Murali T, Tyson JJ (2015). From start to finish: computational analysis of cell cycle control in budding yeast. Syst Biol Appl.

